# Intrinsically disordered proteins and their (disordered) proteomes in neurodegenerative disorders

**DOI:** 10.3389/fnagi.2015.00018

**Published:** 2015-03-02

**Authors:** Vladimir N. Uversky

**Affiliations:** ^1^Department of Molecular Medicine, USF Health Byrd Alzheimer's Research Institute, Morsani College of Medicine, University of South FloridaTampa, FL, USA; ^2^Biology Department, Faculty of Science, King Abdulaziz UniversityJeddah, Saudi Arabia; ^3^Institute for Biological Instrumentation, Russian Academy of SciencesPushchino, Russia; ^4^Laboratory of Structural Dynamics, Stability and Folding of Proteins, Institute of Cytology, Russian Academy of SciencesSt. Petersburg, Russia

**Keywords:** intrinsically disordered proteins, protein-protein interaction, posttranslational modification, neurodegenerative diseases, proteome

The recent years have witnessed a rise in the number of intrinsically disordered proteins (IDPs), also known as hybrid proteins, which possess both structured domains and biologically important intrinsically disordered protein regions (IDPRs). These proteins challenge the “one sequence—one structure—one function” concept by demonstrating that the lack of stable tertiary and/or secondary structure does not preclude proteins from being biologically active (Wright and Dyson, 1999; Uversky et al., 2000; Dunker et al., 2001; Tompa, 2002; Uversky and Dunker, 2010). Both ordered and disordered/hybrid proteins tend to misfold under certain conditions, and the aggregation that typically accompanies protein misfolding is associated with the pathogenesis of several human diseases, particularly those that originate from the deposition of protein aggregates in a variety of organs and tissues (Kelly, 1998; Bellotti et al., 1999; Dobson, 1999; Uversky et al., 1999a,b; Rochet and Lansbury, 2000; Uversky and Fink, 2004; Gasperini et al., 2012; Moreau and King, 2012; Safar, 2012; Walker and LeVine, 2012; Cuanalo-Contreras et al., 2013; Mulligan and Chakrabartty, 2013; Hipp et al., 2014). Misfolding and aggregation of IDPs/IDPRs are especially common in neurodegeneration (Uversky, 2010, 2014a; Breydo and Uversky, 2011). An incomplete list of human neurodegenerative diseases associated with IDPs/IDPRs is provided below. This list shows that some IDPs are involved in several diseases and that some neurodegenerative diseases are associated with misbehavior of several IDPs/IDPRs. The list includes Alzheimer's disease [AD, where the deposition of amyloid-β, tau-protein, and α-synuclein fragment NAC (Glenner and Wong, 1984; Ueda et al., 1993) takes place]; Niemann-Pick disease type C, subacute sclerosing panencephalitis, argyrophilic grain disease, myotonic dystrophy, and motor neuron disease with neurofibrillary tangles (NFTs) (accumulation of tau-protein in the form of NFTs, Lee et al., 1991); Down's syndrome (nonfilamentous amyloid-β deposits, Wisniewski et al., 1985); Parkinson's disease (PD), dementia with Lewy body (LB), diffuse LB disease, LB variant of AD, multiple system atrophy (MSA), and Hallervorden-Spatz disease [all characterized by the deposition of α-synuclein in a form of LB, or Lewy neurites (LNs), Dev et al., 2003]; amyotrophic lateral sclerosis (ALS) and frontotemporal lobar degeneration (FTD) [both characterized by the presence of the cytoplasmic inclusions rich in transactive response element DNA-binding protein of 43 kDa (TDP43) (Nass et al., 2012; Barmada et al., 2014)]; aberrant accumulation of the wild type and mutated fused in sarcoma/translocated in liposarcoma (FUS/TLS) in the cytosol of voluntary motor neurons in sporadic and familial ALS (Pokrishevsky et al., 2012; Sreedharan and Brown, 2013; Ajroud-Driss and Siddique, 2015); prion diseases (deposition of PrP^SC^, Prusiner, 2001); and a family of polyQ diseases, a group of neurodegenerative disorders caused by the expansion of GAC trinucleotide repeats that code for polyQ in the gene products (Zoghbi and Orr, 1999).

There are several reasons for why IDPs/IDPRs are so common in neurodegenerative diseases. Firstly, these proteins/regions, with their unique structural plasticity, conformational adaptability, ability to react quickly in response to changes in their environment, and their binding promiscuity, are abundantly involved in various signaling, regulation, and recognition processes, and play diverse roles in the modulation and control of the functions of their binding partners (Dyson and Wright, 2005; Oldfield et al., 2008; Uversky and Dunker, 2010; Cozzetto and Jones, 2013; Ferreon et al., 2013). Secondly, the biological activities of IDPs/IDPRs are under tight control and are regulated by means of extensive posttranslational modifications (PTMs), such as phosphorylation, acetylation, glycosylation (Collins et al., 2008; Uversky and Dunker, 2010; Kurotani et al., 2014; Pejaver et al., 2014), and by alternative splicing (Romero et al., 2006; Buljan et al., 2012, 2013; Uversky, 2014b). Thirdly, many IDPs and hybrid proteins are able to interact with a large number of unrelated partners, thereby serving as hubs in cellular protein-protein interaction networks (Dunker et al., 2005; Uversky et al., 2005; Dosztanyi et al., 2006; Ekman et al., 2006; Haynes et al., 2006; Patil and Nakamura, 2006; Singh et al., 2007; Singh and Dash, 2007). Lastly, IDPs/IDPRs are often able to fold differently while interacting with different binding partners (Dyson and Wright, 2005; Oldfield et al., 2008; Hsu et al., 2013).

Since IDPs/IDPRs play a number of crucial roles in numerous biological processes, it is not surprising that some of these proteins are related to the pathogenesis of human disease, and to neurodegenerative processes in particular. In fact, dysregulation and misfolding of the otherwise tightly controlled IDPs/IDPRs can result in their dysfunction, ultimately leading to the development of life-threatening pathological conditions. Mutations and/or changes in the environment may reduce the capability of a protein to recognize proper binding partners, leading to the formation of nonfunctional complexes and aggregates (Uversky et al., 2005). The topic of the involvement of IDPs/IDPRs in neurodegeneration was covered in recent reviews (Uversky, 2010, 2014a; Breydo and Uversky, 2011). Table 1 clearly shows that some individual proteins involved in the pathogenesis of human neurodegenerative diseases are either completely disordered or contain long disordered regions. Table 1 also illustrates that neurodegeneration-related IDPs are characterized by astonishing binding promiscuity, as they are able to interact with a large number of unrelated partners. It is worth noting that the numbers shown in Table 1 correspond to the minimal interactomes, or “first interaction shells”; i.e., they correspond to the numbers of proteins directly interacting with a given neurodegeneration-related protein. Many of the proteins in such “first interaction shells” interact with other proteins, thereby generating a broadened “second interaction shell.”

**Table 1:** **IDPs and associated neurodegenerative diseases**.

**Protein (number of residues)**	**Disease(s)**	**Disorder by prediction (%)^a^**	**Number of binding partners on BioGrid^b^**
Aβ (42)	Alzheimer's disease	16.9 (28.6)	1975 (for the Aβ precursor protein)
	Dutch hereditary cerebral hemorrhage with amyloidosis		
	Congophilic angiopathy		
Tau (758)	Tauopathies	77.6 (99.1)	73
	Alzheimer's disease		
	Corticobasal degeneration		
	Pick's disease		
	Progressive supranuclear palsy		
Prion protein (231)	Prion diseases	55.8 (61.0)	60
	Creutzfeld-Jacob disease		
	Gerstmann- Sträussler -Schneiker syndrome		
	Fatal familial insomnia		
	Kuru		
	Bovine spongiform encephalopathy		
	Scrapie		
	Chronic wasting disease		
α-Synuclein (140)	Synucleinopathies	90.7 (37.1)	416
	Parkinson's disease		
	Lewy body variant of Alzheimer's disease		
	Diffuse Lewy body disease		
	Dementia with Lewy bodies		
	Multiple system atrophy		
	Neurodegeneration with brain iron		
	accumulation type I		
β-Synuclein (134)	Parkinson's disease	87.3 (52.2)	16
	Diffuse Lewy body disease		
γ-Synuclein (127)	Parkinson's disease	100 (56.8)	26
	Diffuse Lewy body disease		
TDP43 (414)	Amyotrophic lateral sclerosis and frontotemporal lobar degeneration	57.3 (35.8)	286
FUS (526)	Amyotrophic lateral sclerosis	90.7 (72.6)	105
Huntingtin (3144; polyQ tract: 16–37 Qs in norm; >38 Qs in pathology)	Huntington's disease	35.5 (30.4)	193
*DRPLA protein*	Hereditary dentatorubral-pallidoluysian atrophy	89.5 (84.2)	98
(1185; polyQ tract: 7–23 Qs in norm; 49–75 Qs in pathology)			
Androgen receptor (919; polyQ tract: 15–31 Qs in norm; 40–62 Qs in pathology)	Kennedy's disease or X-linked spinal and bulbar muscular atrophy	53.9 (46.7)	219
Ataxin-1 (816; polyQ tract: 6–39 Qs in norm; 41–81 Qs in pathology)	Spinocerebellar ataxia 1	76.8 (73.4)	254
	Neuronal intranuclear inclusion disease		
Ataxin-2 (1312; polyQ tract: 22–31 Qs in norm; >32 Qs in pathology)	Spinocerebellar ataxia 2	93.8 (76.9)	44
Ataxin-3 (376; polyQ tract: 12–40 Qs in norm; 55–84 Qs in pathology)	Spinocerebellar ataxia 3	52.1 (47.1)	76
P/Q-type calcium channel α1A subunit (2505; polyQ tract: 4–16 Qs in norm; 21–28 Qs in pathology)	Spinocerebellar ataxia 6	53.0 (49.3)	94
Ataxin-7 (892; polyQ tract: 4–35 glutamines in norm; 36–306 glutamines in pathology)	Spinocerebellar ataxia 7	89.5 (70.2)	83
TATA-box-binding protein (339; polyQ tract: 25–42 glutamines in norm; >42 glutamines in pathology)	Spinocerebellar ataxia 17	53.9 (52.5)	145
Glial fibrillary acidic protein (432)	Alexander's disease	82.4 (68.5)	33
DNA excision repair protein ERCC-6 (1493)	Cockayne syndrome	56.8 (47.8)	40
Survival motor neuron protein (294)	Spinal muscular atrophy	69.7 (60.2)	186

a *Disorder was predicted by two predictors, PONDR^®^ VSL2 and VLXT (given in parenthesis), respectively. PONDR^®^ VSL2 was chosen because of its high accuracy, whereas PONDR^®^ VLXT was chosen because this predictor was shown to be very sensitive for the presence of molecular recognition features, which are disordered polypeptide segments that are predicted to acquire secondary structure upon forming complexes with binding partners*.

b *Interactivity of neurodegeneration-related proteins was evaluated by BioGrid^3.2^ (Chatr-Aryamontri et al., 2013, 2015)*.

Neurodegeneration-related proteins are intrinsically disordered hubs with highly extended proteomes. Due to the overall abundance of IDPs, at least some of these binding partners are disordered proteins themselves. This gives an interesting twist to the involvement of IDPs in neurodegeneration, since the neurodegeneration-related IDPs listed in Table 1 are not only abundantly found in neurodegenerative diseases, but are also regulated and controlled by other IDPs. A few related examples are provided below to illustrate the ensuing “chaos to control chaos” concept.

The first example deals with the proteins directly involved in the control of normal cellular proteostasis: protein chaperones. Protein chaperones form a specific network that constitutes the major component of the cellular quality control (McClellan et al., 2005; Bukau et al., 2006; Leidhold and Voos, 2007; Witt, 2010). Computational studies revealed that ~40% of chaperones' residues are located within the disordered regions, with ~15% of the residues falling within long IDPRs exceeding 30 consecutive residues (Tompa and Csermely, 2004). Studies have shown that many neuroprotective chaperones/co-chaperones are either completely disordered or possess long IDPRs (Uversky, 2011). The illustrative examples of these disordered/hybrid chaperones include Hsp70 (C-terminal part of substrate binding domain and lid domain); members of the human DnaJ homolog subfamilies A, B, and C; various Hsp70 co-chaperones [Hip, Hsp100, BAG family molecular chaperone regulator 1 L, CHIP/STIP1, Hop (Hsp70/Hsp90-organization protein)]; linker regions in human Hsp90α and Hsp90β; Hsp90 co-chaperones p23 and FKBP52; small heat shock proteins Hsp27/HspB1, HspB2, HspB3, αA-crystallin/HspB4, αB-crystallin/HspB5, Hsp20/HspB6, cvHsp/HspB7, H11/HspB8, HspB9, and outer dense fiber protein 1 (ODF1); human α-, β-, and γ-synucleins; prefoldin subunits. (Uversky, 2011). It was demonstrated that the presence of disorder determines the promiscuity of chaperones, allowing them to act as pliable molecular recognition elements, to wrap misfolded chains, and to participate in disaggregation and local unfolding of the aggregated and misfolded species (Uversky, 2011). Furthermore, disorder plays a number of important roles in the precise orchestration of coordinated actions of chaperones, co-chaperones, and their auxiliary and regulatory proteins, which intricately communicate with each other to form a sophisticated and highly flexible network of malleable guardians (Uversky, 2011).

The second example is related to sirtuins that constitute an important family of regulatory proteins involved in several physiological functions, including control of gene expression, metabolism, and aging (Paraiso et al., 2013). In mammals, there are seven sirtuins (Sirt1 to Sirt7), with members of this protein family being highly expressed in various regions of the brain. Here, sirtuins are involved in cognitive functions and regulate cellular protection against oxidative stress in many neurodegenerative diseases (Gan and Mucke, 2008). For example, in animal PD models, Sirt1 regulates autophagy (Lee et al., 2008), which is responsible for the aggregated α-synuclein clearance (Paraiso et al., 2013), and therefore Sirt1 can reduce α-synuclein aggregation (Zhang et al., 2012). In AD models, the overexpression of Sirt1 modulates the processing of APP by increasing the activity of α-secretase (Bonda et al., 2011), which shifts APP processing toward the non-amyloidogenic Aβ forms (Bonda et al., 2011). Furthermore, Sirt1 deactivation has been associated with increased levels of acetylated and pathogenic phosphorylated forms of tau protein (Min et al., 2010). In HD mouse models, some neuroprotection was reported for Sirt1 overexpression (Jeong et al., 2012). These data show that sirtuins can act as important regulators of several neurodegenerative diseases caused by the misbehavior of several neurodegeneration-promoting IDPs (α-synuclein, tau, Aβ, and huntingtin). Recent *in silico* analysis of the sirtuin family members showed that all these proteins have long disordered arms that play crucial biological roles such as recognition and interaction with other protein molecules (Costantini et al., 2013).

The third example emphasizes the important role of intrinsic disorder in the maintenance of stress granules (SGs) that are potentially related to the pathology of some neurodegenerative diseases (Wolozin, 2012; Bentmann et al., 2013). These SGs are formed as a cellular stress response and are cytoplasmic membrane-less organelles that contain several RNA-binding proteins and RNA molecules that are stalled at the pre-initiation stage. These RNA molecules and binding proteins possess defined cytoprotective function (Bentmann et al., 2013). Importantly, all the major players responsible for the nucleation and maturation of SGs are either IDPs or hybrid proteins containing long, functionally important IDPRs (Uversky et al., 2015). Furthermore, SGs were shown to co-localize with insoluble protein aggregates in many neurodegenerative diseases (Wolozin, 2012). They commonly include RNA-binding proteins related to the pathogenesis of various neurodegenerative diseases, such as TDP-43 and FUS (related to the pathology of ALS and FTD), SMN (related to SMA pathology), ataxin-2 (related to SCA2), optineurin (related to primary open angle glaucoma and ALS12), and angiogenin (involved in ALS9 pathology). They may also contain, and be regulated by, proteins such as tau (Wolozin, 2012), PrP^SC^ (Goggin et al., 2008), huntingtin (Waelter et al., 2001), and some other Q/N-rich proteins (Furukawa et al., 2009).

The last example emphasizes the role of the tubulin polymerization promoting protein (TPPP)/p25 in regulating α-synuclein aggregation in multiple system atrophy (MSA). The α-synuclein-containing glial cytoplasmic inclusions are the neuropathological hallmark of MSA. Recent studies revealed that the pathological aggregation of α-synuclein in oligodendroglia is dramatically accelerated by TPPP/p25 (Hasegawa et al., 2010), which is a highly disordered and widely expressed protein that possesses multiple PTM sites, and is involved in a multitude of interactions with unrelated partners. Structural analysis revealed that TPPP/p25 is a typical IDP that partially folds as a result of Zn^2+^ binding (Zotter et al., 2011).

## Conclusions

Human neurodegenerative diseases are commonly associated with the misbehavior of IDPs and hybrid proteins containing ordered domains and IDPRs. This link between intrinsic disorder and neurodegeneration is determined by the specific structural and functional features of IDPs/IDPRs, which are some of the major cellular regulators, recognizers, and signal transducers. The normal functionality and pathological misbehavior of IDPs/IDPRs are both modulated by various PTMs and alternative splicing, with dysregulation of these regulatory mechanisms being a crucial contributing factor to dysfunction of related proteins and, consequently, to neurodegeneration. IDPs/IDPRs are promiscuous binders that can fold differently upon interaction with different binding partners. Furthermore, proteomes of such neurodegeneration-related IDPs/IDPRs are vast and contain numerous regulatory IDPs. Therefore, pathogenesis of neurodegenerative diseases is commonly driven by the dysfunction of corresponding IDPs, and the normal and pathogenic behavior of such disease-related IDPs are controlled by other IDPs.

### Conflict of interest statement

The author declares that the research was conducted in the absence of any commercial or financial relationships that could be construed as a potential conflict of interest.
